# *PITX2* and *PANCR* DNA methylation predicts overall survival in patients with head and neck squamous cell carcinoma

**DOI:** 10.18632/oncotarget.12417

**Published:** 2016-10-03

**Authors:** Verena Sailer, Emily Eva Holmes, Heidrun Gevensleben, Diane Goltz, Freya Dröge, Luka de Vos, Alina Franzen, Friederike Schröck, Friedrich Bootz, Glen Kristiansen, Andreas Schröck, Dimo Dietrich

**Affiliations:** ^1^ Weill Medical College of Cornell University and New York Presbyterian Hospital, Department of Pathology and Laboratory Medicine, New York, NY, USA; ^2^ Weill Medical College of Cornell University and New York Presbyterian Hospital, Englander Institute for Precision Medicine, New York, NY, USA; ^3^ Institute of Pathology, University Hospital of Bonn, Bonn, Germany; ^4^ Department of Otorhinolaryngology, University Hospital Essen, University of Duisburg-Essen, Essen, Germany; ^5^ Department of Addictive Disorders and Addiction Medicine, LVR Hospital Bonn, Bonn, Germany; ^6^ University Hospital Bonn, Department of Otolaryngology, Head and Neck Surgery, Bonn, Germany

**Keywords:** PITX2, biomarker, head and neck squamous cell carcinoma, DNA methylation, PANCR

## Abstract

**Background:**

Squamous cell carcinoma of the head and neck region (HNSCC) is a common malignant disease accompanied by a high risk of local or distant recurrence after curative-intent treatment. Biomarkers that allow for the prediction of disease outcome can guide clinicians with respect to treatment and surveillance strategies. Here, the methylation status of *PITX2* and an adjacent lncRNA (*PANCR*) were evaluated for their ability to predict overall survival in HNSCC patients.

**Results:**

*PITX2* hypermethylation was associated with a better overall survival (hazard ratio, HR = 0.51, 95%CI: 0.35-0.74, p<0.001), while *PANCR* hypermethylation was significantly associated with an increased risk of death (HR = 1.64, 95%CI: 1.12-2.39, p=0.010).

**Methods:**

Quantitative, methylation-specific real-time PCR assays for *PITX2* and *PANCR* were employed to measure bisulfite-converted DNA from formalin-fixed, paraffin-embedded (FFPE) tissues in a cohort of 399 patients with localized or locally advanced HNSCC who received curative-intent treatment (surgery with optional adjuvant radiochemotherapy or definite radiochemotherapy).

**Conclusions:**

*PITX2* and *PANCR* methylation status were shown to be independent predictors for overall survival in HNSCC patients. Tissue-based methylation testing could therefore potentially be employed to identify patients with a high risk for death who might benefit from a more radical or alternative treatment.

## INTRODUCTION

Cancer of the head and neck region is a frequent and worldwide health burden with 61.760 estimated new cases and 13.190 estimated deaths in the United States for 2016 alone [1]. More than 90% of these tumors are squamous cell carcinomas (HNSCC) occurring in the oral and nasal cavity, pharynx, and larynx [2]. Tobacco smoking and alcohol consumption are well known carcinogenic risk factors, however, during the past two decades human papilloma virus (HPV) infections have also been related to oropharyngeal carcinomas. HPV-related tumors are a distinct entity that is associated with an overall better prognosis [3–5], and an etiological link between infection with high risk HPV serotypes 16 and 18 and the development of oropharyngeal tumors is well documented [6]. HPV-related tumors show an activation of the PI3Kinase pathway through activating mutations as well as truncating mutations and deletions of *TRAF3*, whereas smoking-related tumors predominantly harbor inactivating *TP53* and *CDKN2A* mutations. Genomic instability of HNSCC in general is demonstrated by a large number of copy number alterations [7].

Two-thirds of HNSCC patients are diagnosed at an advanced stage (Stage III to IVA/B) [8]. Treatment modalities e.g. surgery, radiotherapy, and chemotherapy are often combined in a multimodality approach resulting in satisfactory loco-regional disease control [9]. Five year survival rates range between 40-60% [10]. Up to 60% of patients develop loco-regional recurrence and up to 30% will have distant failure. Recurrent or metastatic cancer represents the onset of a highly aggressive disease with dismal survival rates [11, 12].

As for other tumors, biomarkers are urgently needed to identify patients that are at risk of recurrent disease and may therefore benefit from a more aggressive first line treatment or intensified surveillance. Current strategies rely solely on established clinico-pathological parameters, e.g. tumor-stage and lymph node involvement, to detect high-risk patients. Unfavorable molecular features are presently not considered to decide for a neoadjuvant therapy or to define follow-up examinations. With the advance of immunotherapeutic agents like the immune checkpoint modulators pembrolizumab and nivolumab, new therapeutic options are available for an effective treatment of HNSCC patients; even in the setting of metastatic disease [13, 14]. This necessitates the identification of high risk patients in order intensify surveillance and enable the early initiation of palliative treatment. Furthermore, adjuvant and neoadjuvant immunotherapy protocols, which are under investigation but not yet established, might be considered for patients who are at high risk of disease-related death.

In HNSCC, HPV status has been shown to be associated with better overall survival after platinum-based radiochemotherapy [15]. Independent of HPV status, a high expression of intratumoral CD8-positive T-lymphocytes can be employed as a prognostic maker for prolonged overall survival [16]. Comprehensive molecular profiling has revealed an abundance of molecular variations in HNSCC, but the majority of alterations lack prospective validation as biomarkers in large patient cohorts [17, 18].

A promising approach to identify biomarkers in any tumor is the investigation of gene methylation status and its correlation with clinical parameters. Epigenetic deregulation, indicated by aberrant hypo- and hypermethylation, is a frequent event in human cancer and plays a key role in carcinogenesis [19, 20]. Several attempts have been made to employ methylation status as a predictive or prognostic biomarker. *MGMT* promoter methylation predicts progression-free survival in patients with glioblastoma multiforme undergoing therapy with temozolomide. So far, *MGMT* methylation is the only routinely tested methylated biomarker in clinical practice [21, 22]. Recently, QIAGEN (Hilden, Germany) and Therawis (Munich, Germany) have embarked on a cooperation to market a *PITX2* methylation assay for treatment stratification in breast carcinoma patients.

The homeobox gene *PITX2* is located on chromosome 4q25 and encodes four isoforms (PITX2A, PITX2B, PITX2C, PITX2D) of bicoid transcription factors involved in the development of anterior structures [23]. Mutations in *PITX2* are responsible for Axenfeld-Rieger syndrome Type I, a disorder affecting the development of teeth, eyes, and abdominal structures [24]. *PITX2* gene expression is regulated by the Wnt pathway and interacts with the potent transcriptional activator and mediator of cell adhesion β-catenin as well as promoting cell growth via Cyclin A1 and D2 [25, 26]. Its contribution to tumorigenesis has not been fully elucidated and may depend on the tumor type according to its role as tissue specific transcription factor. *PITX2* hypermethylation, among others, has been found in breast carcinoma and acute myeloid leukemia [27–31]. In prostate cancer, hypermethylation is associated with a significant risk of disease progression [32, 33]. In contrast, *PITX2* overexpression suggestive of low methylation may result in tumor progression in ovarian and thyroid carcinomas [34]. In patients with non-small cell lung cancer (NSCLC), *PITX2* DNA hypomethylation was associated with an increased risk of disease progression [35].

Additionally, non-coding RNAs (ncRNA) have been reported as regulators of gene expression. Even though these RNA strands – representing the vast majority of the transcriptome – are typically not translated into proteins they significantly contribute to cell biology and development regulation [36–39]. Dysregulation of these complex mechanisms can result in a variety of diseases from neurodegenerative disorders to cancer [40]. ncRNAs are divided into two categories: small ncRNAs, like the well-studied microRNAs, and long non-coding RNAs (lncRNA), that are more than 200 nucleotides in length [41]. Recently, *PANCR*, a lncRNA adjacent to the *PITX2* gene, has been identified. It regulates expression of the splice variant PITX2c in cardiomyocytes [42]. So far, the methylation status of *PANCR* has not been described in any disease.

Given the promising, albeit conflicting, data about *PITX2* methylation and risk of disease progression in cancer, the methylation status of *PITX2* and *PANCR* and its potential function as a prognostic biomarker was investigated in a well annotated HNSCC cohort.

## RESULTS

### Design and validation of the PITX2 and PANCR methylation assay

**Figure 1:**
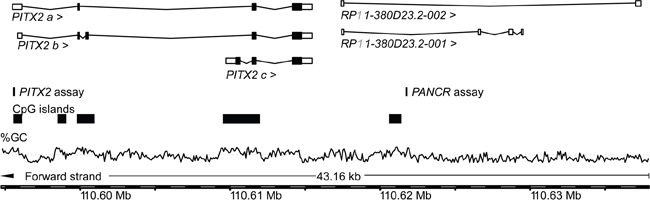
Genomic locations of the *PITX2* and *PANCR* qPCR assays The information was taken from Ensembl Homo sapiens version 82.38 (GRCh38.p3). The GC content [%] is shown with the dashed line indicating 50% GC.

The genomic location of both *PITX2* and *PANCR* assays are shown in Figure 1. The *PITX2* methylation quantitative methylation-specific real-time PCR (qMSP) assay used in the presented study has previously been described in two studies [43, 44]. Methylation analysis is by necessity a quantitative approach, since normal tissue can exhibit different levels of methylation. Thus, a highly accurate quantitative assay is required. The analytical performance of the *PITX2* assay has been verified earlier [43, 44].

**Figure 2:**
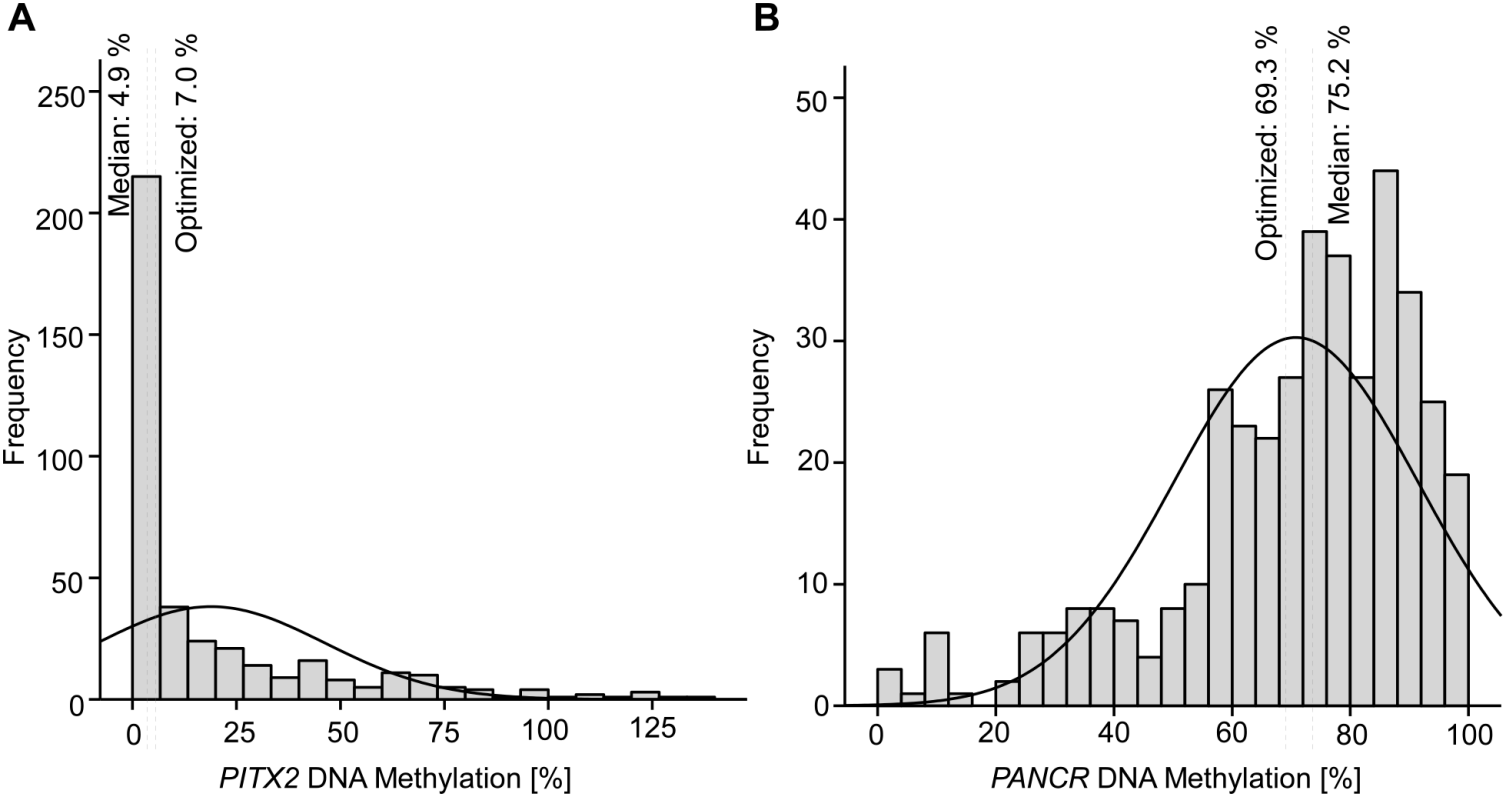
Histogram showing the distribution of *PITX2* A. and *PANCR* B. values in tumor samples from HNSCC patients DNA methylation is depicted in 5% increments on the x-axis. Methylation cut-offs used to dichotomize into low and high DNA methylation was achieved by using median values (Cut-offs for *PITX2*: median = 4.9%, optimized = 7.0%; and *PANCR*: median = 75.2%, optimized = 69.3%).

FFPE samples from 399 patients with HNSCC were analyzed for *PITX2* and *PANCR* methylation. Valid quantification was obtained from 385 (*PITX2*) and 394 (*PANCR*) samples. The distribution of DNA methylation in 5% increments for *PITX2* (Figure 2A) and *PANCR* (Figure 2B) is depicted in histograms. The majority of samples for *PITX2* showed DNA methylation between 0% and 10% (median: 4.9%), whereas *PANCR* was highly methylated with a median of 75.2%. Mean methylation amounted to 18.8% for *PITX2* and 70.8% for *PANCR*.

**Figure 3:**
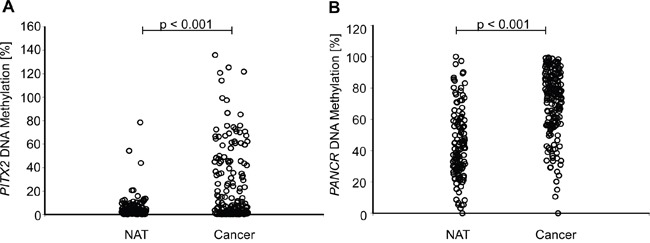
Comparison of *PITX2* A. and *PANCR* B. methylation in tumor and normal adjacent tissue (NAT) HNSCC tumor tissue showed a significantly higher methylation of *PITX2* and *PANCR* compared to the surrounding tissue.

The comparison of *PITX2* and *PANCR* DNA methylation in tumor tissue and normal adjacent tissue (NAT) revealed a significantly higher frequency of hypermethylation in cancer tissue (p < 0.001, Figure 3A, 3B).

### PITX2 and PANCR hypermethylation is associated with clinicopathologic parameters

Patients characteristics were obtained for the majority of patients and were in concordance with reported demographic and clinical data of HNSCC patients [45]. A detailed description of the cohort can be found in Table 1.

**Table 1:** Clinicopathological data and their association/correlation with *PITX2* and *PANCR* methylation in the cohort comprised of surgical specimens from 399 HNSCC patients

Characteristic	No. (%) of Patients	Median *PITX2* Methylation (%)	*p*-value	Median *PANCR* Methylation (%)	*p*-value
All Patients	399 (100)	4.9		75.2	
**Sex**			0.24		0.65
Female	93 (23.3)	4.1		74.0	
Male	306 (76.7)	5.6		75.6	
**Age (Years)**			0.75		0.10
Mean	62.4				
Median	62				
n ≤ Median	168 (42.1)	4.3		75.2	
n > Median	155 (38.8)	5.0		75.4	
Unknown	76 (19.0)				
**Smoking Status**			0.048*		0.17
Non-Smoker	29 (7.3)	13.6		70.7	
Smoker	230 (57.6)	4.5		76.0	
Unknown	140 (35.1)				
Pack Years			0.012*		0.14
(< 40)	122 (30.6)	12.6		72.3	
(> 40)	72 (18.0)	4.5		75.2	
Unknown	205 (51.4)				
**Alcohol Consumption**			0.34		0.10
Never	71 (17.8)	10.4		69.2	
Occasionally	40 (10.0)	4.0		74.2	
Abuse	70 (17.5)	2.2		78.4	
Unknown	218 (54.6)				
**Tumor Site**			0.002*		0.003*
Oral Cavity	68 (17.0)	2.5		74.1	
Oropharynx	159 (39.8)	6.7		79.1	
Hypopharynx	34 (8.5)	1.9		76.1	
Larynx	117 (29.3)	8.9		71.7	
Unknown	21 (5.3)				
**pT**			0.75		0.24
T1/T2	199 (49.9)	5.5		72.3	
T3/T4	141 (35.4)	4.7		76.3	
Unknown	59 (14.8)				
**pN**			0.22		<0.001*
pN neg	169 (42.4)	5.3		72.5	
pN pos	203 (50.9)	4.3		78.8	
Unknown	27 (6.8)				
**Vascular Invasion (V)**					
V neg	202 (50.6)	4.9	0.95	76.0	0.90
V pos	24 (6.0)	4.9		75.6	
Unknown	173 (43.4)				
**p16**			<0.001*		0.004*
Negative	241 (60.4)	3.0		73.8	
Positive	60 (15.0)	39.4		81.3	
Unknown	98 (24.6)				
**Grade**			0.62		0.18
1	7 (1.8)	4.6		74.2	
2	199 (49.9)	4.9		74.4	
3	107 (26.8)	6.3		77.4	
Unknown	86 (21.6)				
**Surgical Margin**			0.53		0.22
Negative	272 (68.2)	4.8		75.0	
Positive	44 (11.1)	5.8		76.8	
Unknown	83 (20.8)				
**Second Tumor**			0.81		0.38
Yes	48 (12.0)	4.3		74.5	
No	201 (50.4)	4.6		77.0	
Unknown	150 (37.6)				

Mann-Whitney U test for sex, smoking status, pT, p16, second tumor, surgical margin, grade; One-Way ANOVA for alcohol consumption, tumor site; Spearman's correlation for age, pack years, pN

* significant feature

Methylation of *PITX2* and *PANCR* was tested as a dichotomized variable with clinico-pathological parameters (p16 expression, nodal status (N), distant metastases (M), vascular invasion (V), and age). A significant correlation was found between p16 expression and *PITX2* and *PANCR* methylation (p< 0.001 and p = 0.004, respectively). Also, the tumor site correlated with *PITX2* methylation (p<0.001). Interestingly, methylation of *PANCR* correlated with lymph node status (p<0.001). No further correlations were observed.

### Hypermethylation of PITX2 and hypomethylation of PANCR is prognostic for overall survival in HNSCC

Cox proportional hazard model analysis was performed with methylation as a continuous variable (Table 2). The quantitative level of *PITX2* was a significant predictor for overall survival (HR = 0.99, 95%CI: 0.98-1.00, p = 0.005). Low methylation levels were associated with a higher risk of death. High *PANCR* methylation, on the other hand, was associated with a significant increase of death (HR = 1.01, 95%CI: 1.00-1.02, p = 0.025). Optimized cut-offs for dichotomizing the data in hyper- and hypomethylated tumor samples were achieved by using a publicly available cut-off finder [46]. The optimization has been carried out with regard to low p-values of the log-rank test. The optimized cut-off was 7.0% for *PITX2* and 69.3% for *PANCR*, respectively (Figure 2). Calculating the Cox proportional hazard model with dichotomized data for both parameters showed that methylation of both loci added significant information about risk of death. *PITX2* hypermethylation resulted in a significantly reduced risk (HR = 0.51, 95%CI: 0.35-0.74, p<0.001), while *PANCR* hypermethylation was associated with a higher risk of death (HR = 1.64, 95%CI: 1.12-2.39, p=0.010).

**Table 2:** Univariate and multivariate Cox proportional hazards model analyses of overall survival including p16 expression, N-category, T-category, vascular invasion (V), tumor site, *PITX2* hypermethylation, and *PANCR* hypermethylation. For dichotomization of *PITX2* and *PANCR* DNA methylation, the optimized cut-off was used

Variable	Univariate		Multivariate	
	HR (95% CI)	p-value	HR (95% CI)	p-value
*PITX2* methylation (continuous variable)	0.99 (0.98-1.00)	0.005*	0.98 (0.96-1.00)	0.001*
*PANCR* methylation (continuous variable)	1.01 (1.00-1.02)	0.025*	1.03 (1.01-1.05)	0.010*
p16 expression (neg. reference)	0.40 (0.20-0.81)	0.010*	0.78 (0.30-2.06)	0.61
N-category	1.28 (1.07-1.53)	0.008*	1.12 (0.81-1.56)	0.49
T-category	1.14 (0.96-1.36)	0.14	1.63 (1.20-2.20)	0.002*
Vascular invasion (V)	3.51 (1.90-6.47)	<0.001*	8.35 (3.70-18.9)	<0.001*
Tumor site	0.86 (0.73-1.01)	0.073	0.66 (0.48-0.90)	0.009*

* significant feature

Multivariate analysis performed with continous variables clearly revealed significant independent information for methylation in both loci, even outperforming p16 expression (Table 2). Methylation analysis continues to be significant when other powerful prognostic parameters like vascular invasion are included in the analysis.

**Figure 4:**
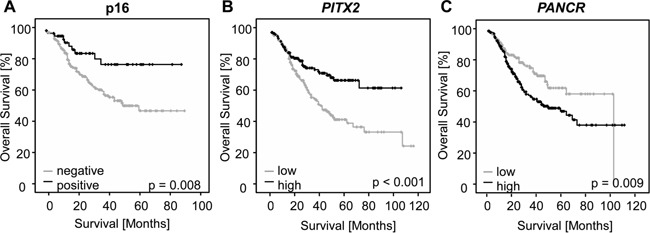
Kaplan-Meier analysis of overall survival in HNSCC patients **A.** Patients (n=284) stratified by p16 expression, a surrogate biomarker for HPV infection. Concordant with published data, patients with HPV-associated (p16-positive) tumors in this study experienced significantly prolonged overall survival. **B** and **C.** Kaplan-Meier analysis of overall survival in HNSCC patients (n = 373) stratified according to the *PITX2* (A) and *PANCR* (B) DNA methylation levels in tumors. Patients with high *PITX2* methylation status survived significantly longer than those with low methylation status. Patients with low *PANCR* methylation status survived significantly longer than those with high methylation status.

Dichotomized data revealed a significantly prolonged overall survival for patients with *PITX2* hypermethylated tumors, as demonstrated in a Kaplan-Meier survival analysis (p=0.002) (Figure 4B). *PANCR* low methylation was significantly associated with prolonged overall survival (p = 0.009) (Figure 4C).

### PITX2 and PANCR methylation is associated with p16 expression

As a surrogate marker of HPV infection, p16 expression was evaluated in 301 primary tumors in a two-tiered manner as either absent or present. A total of 241 tumors (80.1%) were negative, while 60 cases (19.9%) were positive. In concordance with published literature, patients whose tumors presented with p16 expression had a significantly longer survival (p = 0.002, Figure 4A) [47]. Additionally, Cox proportional hazard analysis confirmed that a positive p16 status significantly reduced the risk of death (Hazard Ratio (HR) = 0.40, 95%CI: 0.20-0.81, p = 0.01). High *PITX2* methylation was associated with p16 expression and patients with both p16 positive and *PITX2* hypermethylated HNSCC had a significantly longer survival (p = 0.001). No difference in survival was found when stratifying the data for *PANCR* and p16 status.

## DISCUSSION

HNSCC represents a large health burden worldwide. With regard to survival, only little improvement has been achieved over the last decades [48]. Despite the integration of targeted therapies into the therapeutic portfolio, surgery and radiotherapy are still the mainstay of therapy. A significant number of patients will experience local or distant relapse and some of these may have benefitted from a more aggressive therapy upfront. Tissue-based prognostic biomarkers can be of assistance in stratifying treatment and surveillance intensity. Currently, adjuvant treatment is established only for patients with classical adverse risk factors like pT3/T4 tumors or lymph node metastases [49]. A molecular biomarker could help identifying high-risk patients that would benefit from an adjuvant therapy even without harboring classical risk factors. On the other hand, for patients with high-risk tumors an advantageous molecular profile might prompt omission of adjuvant therapy. However, the molecular mechanisms are incompletely understood therefore the possibility that *PITX2* and *PANCR* status might add to radiosensitivity cannot be ruled out.

A subgroup of patients with both classical and molecular adverse risk factors may benefit from intensifying treatment by adding immunotherapeutic agents upfront. The immune checkpoint inhibitor pembrolizumab has recently been approved by the Food and Drug Administration (FDA) for the treatment of patients with recurrent or metastatic disease after chemotherapy failure. Pembrolizumab is currently being investigated in combination with radiation and chemotherapy and could be part of an adjuvant or neoadjuvant regime in the future (ClinicalTrials.gov Identifiers: NCT02641093, NCT02777385). Promising results in terms of overall survival have also been demonstrated for the PD-1 antibody nivolumab [50].

The findings of the present study show that *PITX2* and *PANCR* methylation status is associated with overall survival. HNSCC patients with tumors harboring a low *PITX2* methylation status have a significantly reduced overall survival. Considering the important contribution of *PITX2* to cell cycle regulation, low methylation may result in augmented transcriptional activity of *PITX2* and subsequent tumor growth. Consequently, high methylation levels may lead to *PITX2* silencing and thus, inhibition of its downstream target effects.

*PITX2* methylation has been investigated in other tumors as well. In contrast to the findings in the present study hypermethylation in breast and prostate cancer is associated with poor outcome [28–30, 32, 33]. In prostate cancer, *PITX2* is an upstream regulator of a key transcription factor, the androgen receptor (AR). Hypermethylation of the *PITX2* promoter in postate cancer cell lines might result in dysregulation and activation of this pathway, in particular in advanced disease [51]. In NSCLC, particular the squamous cell subtype however, hypermethylation is associated with an improved overall survival [43]. Both HNSCC and NSCLC are smoking-related, hormone-independent tumors harboring a high frequency of alterations in the *TP53* pathway [7, 52]. Further research is needed to elucidate the interaction between *PITX2*, *PANCR*, and the *TP53* pathway.

Interestingly, a significant correlation between p16 expression and tumor site and high methylation was demonstrated, underscoring that tumorigenesis in HPV-positive tumors does not predominantly involve the Wnt/μ-Catenin pathway. In contrast a high PITX2 protein expression, suggestive of a low or unmethylated *PITX2* gene, is found in colorectal cancer where the WNT/β-Catenin is predominantly activated due to APC mutations [53]. The same has been demonstrated in other tumors with an aberrant WNT/ß-catenin pathway like esophageal squamous cell cancer [54, 55].

P16 expression has been shown to be prognostic for overall survival in patients with oropharyngeal squamous cell carcinomas [47]. Therefore, it would be interesting to evaluate whether *PITX2* or *PANCR* methylation add additional prognostic information beyond that of p16 status. However, since the number of oropharyngeal tumors in this cohort is too low this question could not be addressed.

Long noncoding RNA has been identified as an important regulator of gene expression both during and after transcription. Dysregulation of lncRNA is implicated in the development of many cancer types [56]. The exact regulatory mechanism, however, is not very well understood and even less is known about epigenetic regulation of lncRNA. The recently described *PITX2* adjacent non-coding RNA (*PANCR*) has so far never been investigated in human cancer and this is the first study to analyze *PANCR* methylation at all. In our study, improved survival was associated with low *PANCR* methylation, however, the transcriptional consequence of *PANCR* methylation is unclear. Further investigation including transcriptomic data is needed to define whether *PANCR* plays a role in HNSCC development and progression. High *PANCR* methylation furthermore augmented the risk of death. It is well established that methylation in the transcriptional start region of a gene may lead to transcriptional repression. Methylation of CpG island in the gene body, on the other hand, can be positively associated with gene expression [57]. Therefore, it is intriguing to hypothesize that the low methylation of *PANCR* occurs at an intragenic CpG island and is associated with a high transcriptional activity of the whole locus, including *PTIX2*.

Interestingly, when compared with the surrounding normal tissue, hypermethylation of both gene loci was significantly more frequent in the tumor tissue. Tumorigenesis in HNSCC has long been established to be subject to a field cancerization, i.e. long-term exposure to noxa results in accumulation of genetic damage in a given anatomic location [58]. In contrast, other alterations like overexpression of the epidermal growth factor receptor (EGFR) are present in tumor-adjacent, morphologically normal mucosa [59].

In general, recurrent disease is diagnosed by radiological studies and/or biopsy. Patients may not qualify for further tumor resection but instead undergo radiotherapy and/or chemotherapy. Thus, tissue for ancillary analysis in addition to clinically ordered tests (immunohistochemistry, whole exome sequencing or targeted gene panels) is very limited. In this study, the often scant tumor tissue of recurrent disease was therefore not tested. However, a stepwise analysis of normal mucosa of high risk/no risk patients, dysplastic epithelium, carcinoma *in situ*, invasive squamous cell cancer, lymph node and distant metastases is needed to fully understand the role of *PITX2* and *PANCR* methylation in development and progression of HNSCC.

In brief, this study that shows that *PITX2* and *PANCR* methylation status are strong predictors of overall survival in HNSCC patients. Tissue-based measurement of both *PITX2* and *PANCR* provides an objective result that can support clinicians in making treatment and surveillance decisions. A transfer of the assay to biopsy samples may allow for decision making prior to surgery or definite radiochemotherapy. However, implementing *PITX2* and *PANCR* methylation status as a biomarker into everyday clinical practice is not yet feasible. A prospective validation in an independent patient cohort is needed to address the overall benefit of these two tissue-based biomarkers.

## MATERIALS AND METHODS

### Patients and ethics statement

The present study was approved by the Institutional Review Board at the University Hospital of Bonn which waved the need for written informed consent. In total, 399 patients with localized or locally advanced HNSCC treated with curative intent at the University Hospital Bonn were included. Tumors presenting with non-squamous histology were not included in the cohort. Overall survival was calculated as time between primary therapy and death. Clinical and pathological parameters like smoking habits and p16 immunohistochemistry, as surrogate marker for HPV infection, were obtained for part of the cohort [60].

### DNA extraction and bisulfite conversion

Samples were obtained from 399 HNSCC patients. For each patient, 2 μm sections from formalin-fixed and paraffin-embedded (FFPE) tissues were stained with hematoxylin and eosin. A board certified pathologist annotated the tumor area and 1-5 cores (0.6 mm in diameter) were punched from each tumor and collected in a 1.5 ml tube (Eppendorf, Hamburg, Germany). Sample lysis, bisulfite conversion, and DNA purification was conducted using the innuCONVERT Bisulfite All-in-one Kit (Analytik Jena, Jena, Germany) [61].

### Quantitative real-time polymerase chain reaction (PCR) assays

The PCR assays were performed using an AB 7500 Fast Real-Time PCR System (Life Technologies Corporation, Carlsbad, CA, USA).

#### PITX2 Duplex-PCR

The *PITX2* DNA methylation was quantified by a methylation-specific real-time PCR as described previously [43, 44]. The thresholds and baselines for analysis were set as follows: 0.015 (threshold *PITX2*), 0.01 (threshold *PITX2-Reference*), 3-20 (baseline). The methylation was calculated as follows by the ΔΔCT method as previously published [43].

#### PANCR assay

The *PANCR* DNA methylation was analyzed by a quantitative methylation (QM) real-time PCR assay. The PANCR-assay was performed in a 20-μl reaction containing 0.4 μM of each primer, 0.3 μM of each detection probe, 0.25 mM each dNTP, 1 x PCR buffer [35 mM Tris (pH 8.4), 6 mM MgCl_2_, 50 mM KCl, 4% Glycerol] and 3 U FastStart *Taq* polymerase (Roche, Mannheim, Germany), 0.006 μl ROX solution (prepared as previously described [43]) and 50 ng bisulfite-converted sample DNA (according to UV quantification). The following temperature profile was used: 15 min at 95°C of initial denaturation, followed by 50 cycles with 60 sec at 56°C (100% ramp rate) and 15 sec at 95°C (75% ramp rate). The thresholds and baselines for analysis were set as follows: 0.01 (threshold, *PANCR-P-M*), 0.01 (threshold *PANCR-P-U*), 3-20 (baseline). The methylation was calculated as follows: ΔCT = ΔCT*_PANCR_*_-P-U_ – ΔCT*_PANCR_*_-P-M_, ΔΔCT = ΔCT_sample_ – ΔCT_calibrator_, m*PANCR* = 100% / (1+2^(ΔΔCT)).

### Statistical analysis

The statistical analyses were performed using SPSS, version 20 (SPSS Inc., Chicago, IL, USA). The relationship between input DNA methylation and measured DNA methylation was assessed by linear regression. Correlation with clinicopathological parameters was evaluated using the Spearman's rho correlation coefficient. Comparisons were performed using the Mann-Whitney U and One-way ANOVA test. Overall survival analyses were conducted by Kaplan-Meier and the hazard ratio was evaluated using Cox proportional hazard model analysis. P-values refer to the log-rank test. P-values lower than 0.05 were considered significant.
